# The Role of HIV Infection in Neurologic Injury

**DOI:** 10.3390/brainsci7040038

**Published:** 2017-04-06

**Authors:** Rossana Scutari, Claudia Alteri, Carlo Federico Perno, Valentina Svicher, Stefano Aquaro

**Affiliations:** 1 Department of Experimental Medicine and Surgery, University of Rome Tor Vergata, Rome 00133, Italy; scutari.rossana@gmail.com (R.S.); claudia.alteri@uniroma2.it (C.A.); cf.perno@uniroma2.it (C.F.P.); valentina.svicher@uniroma2.it (V.S.); 2 Department of Pharmacy, Health and Nutritional Sciences, University of Calabria, Arcavacata di Rende (CS) 87036, Italy

**Keywords:** HIV-1, CNS, Neurocognitive-Disorders

## Abstract

The central nervous system (CNS) is a very challenging HIV-1 sanctuary, in which HIV-1 replication is established early on during acute infection and can persist despite potent antiretroviral treatments. HIV-1 infected macrophages play a pivotal role acting as vehicles for HIV-1 to spread into the brain, and can be the major contributor of an early compartmentalization. HIV-1 infection in CNS may lead to a broad spectrum of neurological syndromes, such as dementia, mild neurocognitive disorders, and asymptomatic impairment. These clinical manifestations are caused by the release of neurotoxins from infected cells (mainly macrophages), and also by several HIV-1 proteins, able to activate cell-signaling involved in the control of cellular survival and apoptosis. This review is aimed at highlighting the virological aspects associated with the onset of neurocognitive disorders and at addressing the novel therapeutic approaches to stop HIV-1 replication in this critical sanctuary.

## 1. Introduction

The central nervous system (CNS) represents one of the most important sanctuaries for HIV-1 [1]. The productive infection of HIV-1 in this site is primarily supported by resident macrophages [2], even if astrocytes can also be infected by HIV-1, through the blood brain barrier (BBB), which has been demonstrated by in vitro and in vivo studies [1]. The genetic compartmentalization of viral variants in the CNS suggest that adaptive changes occur in response to unique constraints within the brain microenvironment, including specific target cell populations and immune selection pressures [1].

This infection status often causes neurological symptoms including cognitive and motor dysfunction [3]. In general, this disorder is characterized by a combination of virus-related neurological disorders and neuronal-tissue inflammation [4]. The introduction of antiretroviral therapy (ART) and the use of drugs with great penetration of the blood-brain barrier have drastically reduced the incidence of these complications [5,6,7]. However, since not all anti-HIV drugs are able to cross the blood-brain barrier with high efficiency, a minimal viral replication still persists that causes neurocognitive deficits [8,9]. Therefore these clinical manifestations still remain an important complication for chronic HIV-infected patients, especially for those that belong to special settings such as children, patients with low adherence, and late presenters [8].

Risk factors such as low CD4^+^ (cell count, high viral load at baseline, low CD4^+^ nadir, HCV-coinfection, drug abuse, and metabolic comorbidities are also associated with a higher incidence of HIV-neurocognitive impairment (cited in the following paragraphs) [4,10,11,12,13,14,15,16,17,18].

## 2. Clinical Aspects and Classification of Neurological Disorders

Despite the overall improvement of outcomes in patients receiving ART, neurocognitive impairments continue to be present. These disorders are characterized by several dysfunctions such as decrease of attention, mood alterations, depression, psychomotor disturbs, alteration in the extrapyramidal movements, and spasticity, and are associated with morphological profiles characterized by atrophy, neuro-degeneration, persistent inflammation with microglial nodules, perivascular lymphocytes cuffing, accumulation of multinucleated cells expressing HIV antigens (probably derived from the fusion of the uninfected and infected perivascular macrophages), demyelinization, and white matter gliosis [4,19,20,21].

Several diagnostic schemes have been proposed over the years for the diagnosis of HIV-associated neurocognitive disorders (HAND). Early diagnostic guidelines emphasized the motor, psychosocial, and behavioral symptoms [22], rather than the severity of the impairment. In 2007 and 2011, these requirements evolved and were defined into what is now known as the Frascati Criteria [23] and the Gisslen criteria [6]. Up to now, the Frascati criteria [23] are the most widely used nosology of HAND and are considered the gold standard in HIV research. Its scheme identifies three severity levels of HAND: asymptomatic neurocognitive impairments (ANI), mild neurocognitive disorders (MND), and HIV-associated dementia (HAD). While ANI is characterized by the presence of cognitive function impairment in at least two domains without interfering with everyday function, with no signs of *delirium* or dementia, MND is characterized by a mild cognitive function impairment slightly interfering with normal daily activation. This status could evolve toward the more serious condition called HAD, which is markedly incompatible with normal day-by-day life [4,18,22].

## 3. Cells Involved in the Pathogenesis of HIV-Associated Neurocognitive Disorders

Neuronal damage is due to the interaction between HIV-1 and different cell types (Table 1). The primary cell targets for HIV infection, in the CNS, are resident macrophages, neurons, and astrocytes [3,24,25]. The role of macrophages is essential in the neurodegeneration process; indeed, these cells are resistant to HIV-1 cytopathic effects and thus can sustain a viral infection for prolonged periods of time [26,27,28,29,30,31,32]. In the CNS, four major types of macrophages were identified: meningeal macrophages, macrophages of the choroid-plexus, perivascular macrophages, and microglia [33,34]. Among them, microglia and perivascular macrophages seem to have a fundamental role in neurological damage [33]. Their role in the inflammatory process is to release viral proteins, inflammatory cytokines, and neurotoxins, and to induce astrocyte differentiation, apoptosis, and the alteration of the normal neurogenesis [3,35,36,37].

Microglial resident cells play an important role in the HAND pathogenesis, contributing to the neurodegenerative events through various mechanisms. In fact, glial cells infected by HIV are able to release factors and toxins which cause damage in neurons and astrocytes [3,38,39]. 

Astrocytes are neuroectodermal-derived cells, important components of the blood–brain-barrier, which support the function and metabolism of neurons, the ionic homeostasis into the CNS, scar formation, control the state of the neuronal synapses by the uptake of neurotransmitters, and tissue repair. They also regulate the immune response in the brain [40,41,42]. Furthermore, astrocytes can support low level replication of HIV, allowing the virus to persist in the central nervous system [43], to establish a latent infection. 

Moreover, in vitro studies have shown that viral factors may induce, in HIV-infected cells, the release of other chemo-attractive factors that recruit monocytes and microglia, thus amplifying the neuronal damage [26,44,45]. Additionally, several cellular factors such as IL-1β, TNF-α, or IFN-γ are able to stimulate and reactivate viral replication in latently infected-cells [2,46,47].

## 4. The role of HIV-1 in Neuronal Damage

### 4.1. The Direct Mechanisms

**Figure 1:**
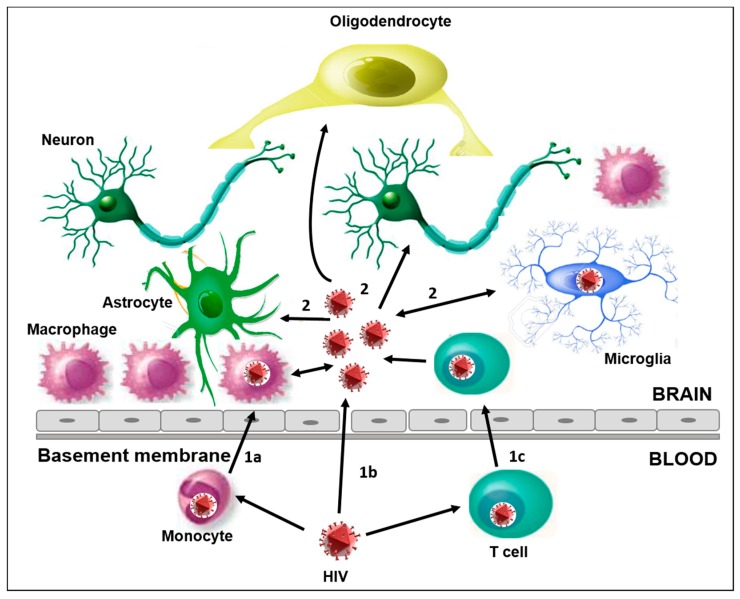
The main characteristics of HIV infection into the CNS. (1) The different ways of HIV-1 entry in the CNS: (1a) the “Trojan horse” mechanism, through HIV-1 infected monocytes that cross the BBB and differentiate into perivascular macrophage; (1b) direct entry, possible in the case of increased permeability due to dysfunctions and/or altered tissue; (1c) the migration into the brain of HIV-1 infected CD4+ T cells. (2) CNS resident cells susceptible to HIV-1 infection are microglia, neurons, astrocytes, and oligodendrocytes. The activation of these cells plays a key role in the release of proinflammatory cytokines, and can amplify the alteration and permeability of the BBB, thus promoting the neuroinvasion of HIV and other viruses.

HIV-1 is able to cross the BBB through three different mechanisms, thereby infecting the CNS (Figure 1). The mechanisms exploited by the virus are the following:

(1) HIV-1 can directly infect endothelial cells, expressing the chemokine receptors involved in HIV-1 entry (CXCR4, CCR3, DC-SIGN) [43,48].
(2) In case of increased permeability due to other dysfunctions and/or altered tissue, the virus may directly enter the BBB [2,49].
(3) In the “Trojan horse” hypothesis, HIV-1 infected-monocytes, leukocytes, and perivascular macrophages crossing the BBB could release viral particles able to infect resident cells like microglia, thus contributing to establishing a persistent infection. This mechanism has also been observed with other retroviruses and lentiviruses and it is probably the main mechanism for HIV penetration into the brain [43]. Several observations suggest that monocytes may be infected before leaving the bone marrow [50]. In particular, an amount of proviral DNA was found in these cells without the expression of viral proteins, thus allowing the dissemination of the HIV-1 infection [50,51]. A relevant role is played by a subset of monocytes (CD14^low^CD16^high^) which tends to increase during HIV-1 infection [34,52,53,54,55,56]. These cells show intermediate characteristics between the monocytes and differentiated cells (macrophage and dendritic cells) [53,55]. They are more permissive to HIV replication, probably due to the lower activity of the host restriction factors than the CD14^high^CD16^low^ cells [51,56], and they can more easily cross the BBB [52,53,54].

Moreover, the release of different viral proteins into the central nervous system could amplify the alteration and permeability of the BBB [57,58] by inducing apoptosis [58,59] and increasing the neuroinvasion of HIV and other viruses [2].

### 4.2. The Indirect Mechanisms

HIV-associated neurologic disorders and neuroinflammation could also depend on three events:

(1) the infiltration of infected monocytes and lymphocytes in the CNS;
(2) the release of viral and cellular factors from these infected cells;
(3) the infection of resident cells by viral particles infiltrating into the CNS or released from infected cells [60].

Cells, such as monocytes and T cells, infected with HIV, have a key role in the release of pro-inflammatory cytokines such as TNF-α and IL-1B, which in turn activate astrocytes and microglia. This cellular activation, in association with perivascular macrophages, is involved in the process of release of neurotoxic factors, such as inflammatory mediators, platelet-derived growth factor (PDGF), nitrogen oxide, and quinolinic acid (QUIN). This leads to dysfunction and neuronal death [2,61].

A previous study has shown that despite effective antiretroviral treatment, cytokines such as CCL2, IL-8, CCL3, CXCL10, IFN-γ, and IL-6 were expressed at higher levels in HIV-1 infected patients with respect to uninfected ones. The higher expression of such cytokines indicates a continuous neuronal inflammation which in turn promotes HAND-associated encephalopathy [1]. Accordingly, Vera and his group have also recently shown the presence of neuroinflammation markers in neuro-asymptomatic HIV-infected patients (despite the effective control of viraemia) [62]. This status is probably related to the impressive microbial translocation from the gut to the bloodstream, causing an extensive inflammation and abnormalities in white matter integrity. These aspects could play an essential role in the pathogenesis of HIV-associated neurocognitive disorders [63].

## 5. The Role of HIV-1 Proteins in HAND

The role of HIV in inducing neuronal damage is deeply described in several works [1,64,65,66,67,68]. Most of these papers focused their attention on the role of the HIV-1 regulatory proteins in this phenomenon (Table 2).

## 6. Tat

In the pathogenesis of HIV-1 infection, a crucial role is played by the regulatory protein Tat [69,70]. Several studies demonstrated that Tat is able to contribute in a different way to neuronal damage. In fact, the exposure of human astrocytoma cells to HIV-1 Tat recombinant protein had a higher level of apoptosis compared to untreated cells [36]. In line with this, the injection at the cerebral level of Tat in mice induces an increase of the Ca^2+^ v1.2 channels, inducing astrogliosis in the cortical region and subsequent death of cortical neurons, microglia, and monocytes [71,72].

Tat seems to also have a role in promoting leukocyte infiltration and invasion [73]. Indeed, this protein induces the expression of both cytokines and chemokines, such as MCP-1/CCL2 (Monocyte chemoattractant protein type 1), of the protein family of CAM (V-CAM 1 and I-CAM1), and the platelet-activating factor (PAF) production [73]. The HIV-1 Tat protein also induces the expression of IL-1β mRNA in a dose-dependent manner [74]. A recent study has shown that Tat can also induce GFAP (glial fibrillary acidic protein) up-regulation, which is known to promote GFAP aggregation and the induction of oxidative stress in astrocytes [75].

In recent findings, the HIV-1 Tat protein also shows a role in up-regulating the Cx43 human gene, involved in the CNS gap junctional communication and promoting, if over-expressed, apoptosis and inflammation, by binding the Cx43 promoter and thus increasing Cx43 mRNA production [76]. In vitro and in vivo studies (based on transgenic mice) have shown that Tat induces a latency excitatory state that may increase the neurotoxic and excitotoxic effects at the presynaptic level [77,78]. Tat may also induce the loss of post-dendritic synapses due to the interference with glutamatergic signaling [79] and with the expression of the dopamine transporter in both the striatum and midbrain [80,81]. 

## 7. Gp120

The envelope glycoprotein gp120, secreted from infected cells, is also involved in HIV-1 pathogenicity [82]. This protein indirectly induces neuronal damage, by stimulating the release of inflammatory cytokines and toxic substances [80]. Moreover, this viral protein might alter the expression of toll-like receptors (TLRs) on astrocytes, favouring the release of TNF-α, IL-6, RANTES/CCL5, and reactive oxygen species, and thus affect the pathogenicity of HIV [82]. Several studies have shown the involvement of gp120 in various processes, such as autophagy. By analysing the markers of autophagy in the brain of gp120-expressing mice, Fields et al. observed a reduced expression of beclin-1, LC3, and neuronal marker MAP2 [83]. In the II, III, and V layers of pyramidal neurons of the midfrontal cortex, this catabolic process is reduced in aged patients compared to young HIV+ and HIV Encephalopathy (HIVE) patients. This leads to the accumulation of altered proteins that can damage the neuronal tissue [83]. In addition, rat cortical neuronal cultures, chronically treated with gp120, showed an increased outward of K^+^ current in a dose-dependent manner [84]. At the neuronal level, the K^+^ increase positively stimulates the apoptotic process. Furthermore, ex vivo studies on the corpus callosum (CC) of HIV infected rats showed that gp120 can stimulate the accumulation of β-APP (a β-amyloid precursor) in axons and induce damage, that can be limited or even stopped with the treatment of CXCR4-antagonists [85]. Thus suggests that the interaction between gp120 and CXCR4 can be important in gp120-mediated CNS damage. 

## 8. Vpr

HIV-1 Vpr (Viral protein R) is an accessory protein that plays different roles during the virus life cycle [86]. This viral protein is released from infected cells, but it can be incorporated in both defective and complete viral particles [60]. This protein is known to increase the release of pro-inflammatory cytokines such as TNF-α, IL-1β, and IL-8 in macrophages, probably acting on the MAPK pathway. It induces apoptosis, probably through the induction of IL1-β and IL-8, known to induce the release of neurotoxins such as matrix metalloproteinases, and to promote cell-cycle and pro-apoptotic proteins [60].

## 9. Nef

HIV-1 Nef is an important viral protein which promotes viral replication and infectivity, by down regulating MHC I receptor expression [87]. In addition, this protein increases the sensibility of astrocytes to hydrogen peroxide [88]. Moreover, this protein promotes astroglial activation and astrogliosis [45]. Nef can also increase the permeabilization of lysosomes causing enzyme release [89], and the subsequent apoptosis of vascular endothelial micro cells (MVECs) [58].

## 10. The Role of Co-Infections

Although the effect of HIV-1 in the CNS has been extensively studied, limited information is available on the effect of co-infection in the CNS.

Epidemiological studies suggest that HIV/HCV co-infection is associated with an accelerated progression of the HIV disease, worsened clinical outcomes, and increased mortality [90,91]. Moreover, individuals co-infected with HIV/HCV most frequently showed neuropsychological deficits, indicative of cognitive disorders [92,93,94].

It has been shown that both viruses are able to increase the permeability of the BBB. In addition, both HIV-1 and HCV have the ability to infect and replicate in microglia, inducing increased expression of proinflammatory cytokines and chemokines, including IL-6 and IL-8.

Vivithanaporn and his group have also shown for the first time that the HCV core protein activates human glia and contributes to neurotoxicity. The in vitro and in vivo aberrant immune activation and neurotoxicity mediated by the HCV core protein were amplified in the presence of the HIV-1 Vpr protein [95]. The data obtained from this study suggest that in individuals co-infected with HIV and HCV, the presence of HCV-proteins causes neuronal damage and perhaps neurocognitive impairment [95].

Recently, Antinori et al. have shown that in HIV/HCV co-infected patients, the levels of HIV-RNA, detectable in both plasma and cerebrospinal fluid (CSF), are associated with increased levels of immunoactivation, neuroinflammation, and neuroinjury biomarkers. Furthermore, the presence of an active HCV replication in CSF seems to be related to increased immunoactivation/inflammation and neuronal injury [96].

Beyond HCV, Herpes Viruses 6, 7 (HHV-6 and HHV-7), and the JC virus (JCV) seem to have a role in accelerating the progression of neurological diseases. For example, HHV-6 stimulates HIV-1 replication into the brain and exacerbates the neurological deficits in children [97]. This herpes virus was also recently described as a causative agent of severe human encephalitis in a HIV-1 infected patient [98]. In the same line of this report, in 2016 the first evidence of a correlation between HHV-7 reactivation and acute myelitis was published [99]. Regarding the polyomaviruses, JCV is known to cause progressive multifocal leukoencephalopathy (PML) in HIV-1 infected individuals [100], resulting from oligodendrocyte lytic infection. Recently, another polyomavirus, HPyV6, seems to have a primary role in favoring demyelination in HIV-1 coinfection [101].

Greater attention has been paid in the last few years to the capacity of microbiomes to influence neurocognitive disorders in HIV-1 infected patients. In particular, the gut microbiome has been implicated in the development and function of brain circuits that support emotion and cognition. Thus, specific modifications may impair this development. In line with this, Perez-Santiago and colleagues showed that the genera of bacteroidetes and firmicutes are more frequently detected in patients with the most severe form of HAND, the HAD, with respect to patients with ANI and MND [102].

## 11. Antiretroviral Therapy and HAND

Great progress has been made in antiretroviral therapy in the last few decades, with a significant improvement in neurological clinical outcomes for HIV-1-infected patients. In particular, in order to reduce the incidence of neurological complications associated with HIV-1 infection [103], international guidelines recommend an immediate first-line treatment regimen for all new diagnosed infected patients [104]. 

However, despite the efficacy of the current antiretroviral therapy in controlling HIV-1, viral replication can still be found in the cerebrospinal fluid in some patients. Indeed, antiretrovirals reach different areas of CSF with significant variability due to the different expression profiles of cellular drug transporters, and the concentrations of some antiretrovirals do not exceed the inhibitory concentration for wild-type HIV replication in CSF (Table 3) [105,106]. The suboptimal concentrations of antiretrovirals within this site also represent the main limitation to achieving HIV-1 eradication from the brain. 

Several factors influence drug concentration in the brain tissue such as molecular weight, lipophilicity, and blood’s protein binding [107,108,109,110]. For example, while entry and integrase inhibitors are able to reach the CNS, the nucleoside/nucleotide reverse transcriptase inhibitors and non-nucleoside reverse-transcriptase inhibitors can only partially cross the BBB. Conversely, the majority of protease inhibitors (PI) are characterized by a medium/low permeability to the BBB [8,111,112,113]. Moreover, several cellular transporters (P-gp, MRP4, and MRP5) can reduce the optimal intracellular concentration of antiretroviral drugs, thus favoring both the emergence of drug-resistant viruses but also their productive infection to other cells [49,58,114,115].

To increase the concentrations of antiretrovirals within this site, new strategies have been identified. For example, the usage of a hypertonic solution of urea or mannitol [50,51] may inhibit drug efflux transport, while nanoparticles and cell-mediated nanoART may confer other key advantages, such as improved blood half-life and bioavailability, higher aqueous stability, and precise delivery [110]. In recent years, different types of nanoparticles have been identified. The most important are: (i) Lipid Nanoparticles (liposomes and solid lipid nanoparticles), that easily cross the barrier due to their lipidic nature [116,117]; (ii) Polymeric Nanoparticles, that exploit the interaction with low density lipoproteins (LDL) receptors on the surface of endothelial cells [111,118]; (iii) Inorganic Nanoparticles, such as small size silica with the addition of polyethylene glycol (PEG) [119], and Gold nanoparticles conjugated with cell-penetrating peptides (CPP) [120]. 

Recently, several groups reported that poly (dl-lactide-co-glycolide) nanoparticles increase the peak concentrations of ritonavir, lopinavir, and efavirenz, all drugs characterized by a low penetration into the CNS. When loaded with Ritonavir, for example, these nanoparticles significantly improved the trans-BBB delivery of the drug in mouse models, inhibiting viral replication in macrophages without inducing neuronal-toxicity [121]. Fiandra and his group reported that poly (dl-lactide-co-glycolide) nanoparticles also increase the peak concentrations of efavirenz [111]. In order to improve the PI penetration into the CNS, two novel non-peptidic PIs, GRL-04810 and GRL-05010, have been also developed. Both these PI are characterized by two additional fluorine atoms that confer to the drugs an increase ability to cross the BBB [8]. However, in order to confirm these findings and the potential role of these compounds in clinical settings, further studies are needed.

In recent years, a CSF Penetration Effectiveness score (CPE) based on pharmacokinetics’ characteristics of various antiretroviral drugs was proposed in order to estimate the efficacy of antiretroviral treatment in the CSF [106]. The effect of this CPE on clinical outcomes in HIV-1 infected individuals was investigated by several papers that showed opposite results [122,123,124]. In particular, while Marra and colleagues demonstrated poorer neurocognitive outcomes in patients with higher CPE scores, other groups found an increased risk of developing dementia in the presence of antiretroviral drugs characterized by good penetration [122,123,124]. These contrasting observations indicate that further studies are required to safely prescribe ART and that regimens characterized by high CPE scores should be carefully chosen in light of the fragile balance between the risk and benefit outcomes.

The acceleration of neurological disorders in the presence of high CPE observed in several clinical studies should be also related to the neuronal toxicity induced by certain antiretroviral drugs, as demonstrated in cell culture and macaques [125,126]. For example, PIs are shown to induce oxidative stress in neuronal cells, while the NNRTI efavirenz caused toxicity in the cortical neuronal cultures of foetal rats [125,126,127]. However, further in vivo studies are needed to confirm the neurotoxicity profiles of these drugs for potential applications in clinical practice. 

## 12. The Compartmentalization of HIV Infection in the CNS

Newly infected individuals typically have homogeneous HIV populations in their blood that evolve during untreated infection to generate diverse viral variants. Compartment-specific selective pressures can subsequently lead to the different evolution of HIV populations in different anatomical sites during the course of infection, including in the CNS. The role of the CNS as an anatomical compartment for HIV infection has been highlighted by several studies [128,129,130]. Harrington and coworkers have elegantly investigated HIV-1 env compartmentalization between cerebrospinal fluid and peripheral blood plasma over all stages of the HIV-1 disease course, including in patients with neurological disorders [129]. Compartmentalization was found only in chronically infected patients with neurological dysfunction, and in patients with HIV-associated dementia (HAD) [129]. This suggests that HIV-1 adaptation and evolution in the CNS milieu play a pivotal role in the onset of neuronal damage. Another interesting study has compared the kinetics of viral decay in cerebrospinal fluid under therapy between asymptomatic patients and in patients with profound immune-depletion and HAD [130]. The authors highlighted a slower viral decay in patients with HAD than in asymptomatic patients; this was not associated with poorer CNS drug penetration, drug resistant viruses in the cerebrospinal fluid, or to the differential presence of the CXCR4-using strains. They proposed a model in which the absence of CD4+ T cells, consequent to profound immunodeficiency, allows peripheral uninfected monocytes to massively migrate in the CNS, to differentiate into perivascular macrophages, and to sustain high levels of HIV-1 replication, thus favouring the onset of a genetically compartmentalized virus in the CNS. 

In line with this model, the HIV-1 load in the CNS seems to be essential for the genesis of neuronal damage [131]. In particular, Soulie and coworkers have found that the ratio of the HIV-1 load in cerebrospinal fluid to the HIV-1 load in plasma is significantly higher in patients with HIV encephalitis than in patients without neurological disorders (11.1 versus 0.7, *p* = 0.0006), proposing this ratio as an instructive marker in predicting HIV encephalitis [131]. By these findings, compartmentalization of HIV-RNA in the CNS has been frequently associated with greater inflammation and worse neurocognitive outcomes.

As previously mentioned, ART has markedly reduced the incidence of HIV-associated dementia. However, the true impact of early ART initiation on HIV-compartmentalization has been only recently investigated. In fact, little is known about the HIV populations persisting in this anatomic compartment during the earliest phase of HIV infection, and especially during suppressive ART. In this regard, a recent study showed that early antiretroviral treatment is associated with a smaller amount of HIV-DNA and a lower molecular diversity of HIV-1 quasispecies in the CSF cells, compared to later ART. Despite this correlation, most participants presented evidence of genetic compartmentalization of the HIV-DNA quasispecies within the CSF with respect to the viral population in blood cells [132], suggesting that early ART is not enough to limit HIV-1 evolution in this compartment. Nightingale and his group have also shown that the discordance between CSF and plasma is more frequently found in patients with low level viremia, and is associated with antiretroviral resistance mutations in the CSF [133], suggesting differential emergence and selection of drug resistance mutations between the CSF and blood during antiretroviral therapy failure, even if at a low viral load.

## 13. Conclusions

Since HIV is able to cross the blood-brain barrier, it can cause infection of the central nervous system. The infection of this compartment involves different cell types but mostly macrophages, that play a crucial role in the neurodegeneration process, releasing neurotoxins, inflammatory cytokines, and viral proteins. Microbial translocation and HCV-coinfection can worsen this pathological condition. However, the optimization of the delivery of antiretroviral drugs into the CNS by nanoformulations has improved the control of HIV-associated neurocognitive disorders and has strongly limited HIV-1 replication. However, it is still insufficient per se to limit viral compartmentalization and to definitely abolish viral residual replication into the CNS.

## Figures and Tables

**Table 1:** **The** role of human cells in HIV-associated neurocognitive disorders.

Cell Type	Infection Type	Effects in the Brain ^a^
Astrocyte	Restricted	Increases BBB permeability; Promotes the migration of monocytes into the brain; Increases the release of intracellular Ca^2+^ and glutamate; Decreases the glutamate uptake; Increases neurotoxins production
Microglia	Productive	Induces the release of viral proteins (gp120, Tat, Vpr); Induces neurotoxins production (inflammatory mediators, PDGF, QUIN); Actives viral replication
Neuron	Restricted	Increases the release of intracellular Ca^2+^; Increases caspase activation; Increases p53 expression
Oligodendrocyte	Restricted	Reduces myelin synthesis; Increases intracellular Ca^2+^ levels; Increases cellular apoptosis
Perivascular Macrophage	Productive	Induces the release of viral proteins (gp120, Tat, Vpr); Induces the neurotoxins production (inflammatory mediators, PDGF, QUIN); Actives viral replication

^a^ Data on the effects of cells in the brain were based on Ref. [3,33,35,37].

**Table 2:** **The** effects of viral regulatory proteins on HAND.

Regulatory Protein	Effects on Hand
**Tat**	- Interacts with Cyclin T1 and CDK9 (69); - Induces the expression of MCP-1/CCL2 (72); - Induces the expression of IL-1β (73); - Induces the expression of GFAP (74); - Up-regulates the Cx43 human gene (75); - Reduces GABA in the cortex (76); - Modulates cellular gene expression; - Increases the expression of GLUT1 in the cortex and hippocampus (76); - Induces the expression of GAC (77); - Reduces the expression of SYN (78); - Increases leukocyte infiltration (72);
**Gp120**	- Stimulates the release of inflammatory cytokines and toxic substances (79); - Reduces the expression of beclin-1, LC3, and MAP2 (82); - Stimulates the accumulation of β-APP (84);
**Vpr**	- Stimulates the release of TNF-α, IL-1β, and IL-8 in macrophages; - Induces the release of neurotoxins (matrix metalloproteinases); - Promotes cell-cycle and pro-apoptotic proteins (59);
**Nef**	- Increases the sensitivity of astrocytes to hydrogen peroxide (87); - Promotes the astroglial activation and astrogliosis (88); - Increases the apoptosis of MVEC (57);

**Table 3:** CNS penetration of the antiretroviral drugs

Drug Class	Drug ^a^	CNS Penetration ^b^
Entry/Fusion inhibitors	ENF	Low
	MVC	High
Integrase strand Transfer inhibitor	RAL	Medium
	EVG	Medium
Nucleoside Reverse Transcriptase inhibitor	ZDV	High
	3TC	Medium
	D4T	Medium
	DDI	Medium
	ABC	Medium
	TDF	Low
	FTC	medium
Non-nucleoside Reverse Transcriptase inhibitor	EFV	medium
	NVP	High
	DLV	High
	ETR	Low
Protease inhibitor	APV	medium
	IDV	medium
	DRV	medium
	RTV	Low
	LPV	Medium
	NFV	Low
	SQV	Low
	ATV	Medium
	FPV	Medium
	TPV	Low

^a^ ENF: Enfuvirtide; MVC: Maraviroc; RAL: Raltegravir; EVG: Elvitegravir; ZDV: Zidovudine; 3TC: Lamivudine; D4T: Stavudine; DDI: Didanosine; ABC: Abacavir; TDF: Tenofovir Disoproxil Fumarate; FTC: Emtricitabine; EFV: Efavirenz; NVP: Nevirapine; DLV: Delavirdine; ETR: Etravirine; APV: Amprenavir; IDV: Indinavir; DRV: Darunavir; RTV: Ritonavir; LPV: Lopinavir; NFV: Nelfinavir; SQV: Saquinavir; ATV: Atazanavir; FPV: Fosamprenavir; TPV: Tipranavir. ^b^ Data on Drug penetration were based on Ref. [111,114].

## Abbreviations:

CCL	Chemokine ligand
CCR3	C-C Chemokine receptor type 3
CD4	Cluster of differentiation 4
CDK	Cyclin-dependent kinase
Cx43	Connexin 43
CXCL	C-X-C chemokine ligand
CXCR4	C-X-C chemokine receptor type 4
DC-SIGN	Cluster of differentiation 209
DNA	Deoxyribonucleic acid
GABA	γ -aminobutyric acid
GFAP	Glial fibrillary acid protein
GLUT1	Glucose transporter 1
gp120	Glycoprotein 120
GRL-04810	nonpeptidic HIV-1 protease inhibitors
GRL-05010	nonpeptidic HIV-1 protease inhibitors
GAC	Glutaminase C
HCV	Hepatitis C virus
HIV-1	Human immunodeficiency virus type 1
IFN-γ	Interferon γ
IL-1β	Interleukin 1β
IL-6	Interleukin 6
IL-8	Interleukin 8
LC3	Microtubule-associated protein 1A/1B-light chain 3
MAP	Microtubule associated protein
MAPK	Microtubule associated protein kinase
MCP-1	Chemokine ligand 2
MRP4	Multidrug resistance protein 4
MRP5	Multidrug resistance protein 5
MVEC	Microvascular endotelial cells
NEF	*Negative Regulatory Factor*
NanoART	Antiretroviral treatment nanoparticle-driven
NNRTI	Non-nucleoside reverse transcriptase inhibitors
PDGF	Platelet-derived growth factor
P-gp	P-glycoprotein
QUIN	Quinolinic acid
SYN	Synaptophysin
TAT	Transactivator HIV protein
TNF-α	Tumor necrosis factor α
VPR	Viral Protein R
β-APP	β amyloid precursor protein
